# Antibody Phage Display Assisted Identification of Junction Plakoglobin as a Potential Biomarker for Atherosclerosis

**DOI:** 10.1371/journal.pone.0047985

**Published:** 2012-10-24

**Authors:** Seraina Cooksley-Decasper, Hans Reiser, Daniela S. Thommen, Barbara Biedermann, Michel Neidhart, Joanna Gawinecka, Gieri Cathomas, Fabian C. Franzeck, Christophe Wyss, Roland Klingenberg, Paolo Nanni, Bernd Roschitzki, Christian Matter, Petra Wolint, Maximilian Y. Emmert, Marc Husmann, Beatrice Amann-Vesti, Wilibald Maier, Steffen Gay, Thomas F. Lüscher, Arnold von Eckardstein, Danielle Hof

**Affiliations:** 1 Institute for Clinical Chemistry, University Hospital Zurich, Zurich, Switzerland; 2 Zurich Center for Integrative Human Physiology (ZIHP), Zurich, Switzerland; 3 Department of Biomedicine, University Hospital Basel, Basel, Switzerland; 4 Institute for Experimental Rheumatology, University Hospital Zurich, Zurich, Zurich, Switzerland; 5 Cantonal Institute for Pathology, Liestal, Switzerland; 6 Department of Cardiology and Cardiovascular Physiology, University Hospital Zurich, Zurich, Switzerland; 7 Functional Genomics Center Zurich (FGCZ), Zurich, Switzerland; 8 Department of Cardiovascular Surgery, University Hospital Zurich, Zurich, Switzerland; 9 Department of Angiology, University Hospital Zurich, Zurich, Switzerland; 10 Competence Center for Systems Physiology and Metabolic Diseases (CC-SPMD), Zurich, Switzerland; University of Freiburg, Germany

## Abstract

To date, no plaque-derived blood biomarker is available to allow diagnosis, prognosis or monitoring of atherosclerotic vascular diseases. In this study, specimens of thrombendarterectomy material from carotid and iliac arteries were incubated in protein-free medium to obtain plaque and control secretomes for subsequent subtractive phage display. The selection of nine plaque secretome-specific antibodies and the analysis of their immunopurified antigens by mass spectrometry led to the identification of 22 proteins. One of them, junction plakoglobin (JUP-81) and its smaller isoforms (referred to as JUP-63, JUP-55 and JUP-30 by molecular weight) were confirmed by immunohistochemistry and immunoblotting with independent antibodies to be present in atherosclerotic plaques and their secretomes, coronary thrombi of patients with acute coronary syndrome (ACS) and macrophages differentiated from peripheral blood monocytes as well as macrophage-like cells differentiated from THP1 cells. Plasma of patients with stable coronary artery disease (CAD) (n = 15) and ACS (n = 11) contained JUP-81 at more than 2- and 14-fold higher median concentrations, respectively, than plasma of CAD-free individuals (n = 13). In conclusion, this proof of principle study identified and verified JUP isoforms as potential plasma biomarkers for atherosclerosis. Clinical validation studies are needed to determine its diagnostic efficacy and clinical utility as a biomarker for diagnosis, prognosis or monitoring of atherosclerotic vascular diseases.

## Introduction

Atherosclerosis with its complications such as acute coronary syndrome (ACS), sudden cardiac death and stroke, is the leading cause of death world-wide. While fatty streaks develop into atheroma and then into complicated atherosclerotic plaques without significant lumen obstruction [1], the patient does not display any symptoms and therefore is often unaware of the risk. In about half of patients, the first manifestation of coronary atherosclerosis is sudden cardiac death or myocardial infarction unheralded by any symptoms [2]. To date, clinical laboratory diagnostics provides important information for cardiovascular risk assessment (notably total-, HDL- and LDL-cholesterol and triglycerides, as well as C-reactive protein (CRP)), acute diagnosis (troponins I or T) and prognosis (e.g. troponins or B-type natriuretic peptides) of coronary artery events [3], [4], [5]. However, the diagnostic and prognostic value of these biomarkers is hampered by their limited sensitivity and/or specificity [6], [7]. Moreover, progression and regression of atherosclerotic vascular diseases can currently be assessed only by expensive imaging techniques [8], [9], which sometimes are even invasive, but not by measuring any reasonably priced disease marker in blood. Hence, there is still a high medical need for novel biomarkers that identify asymptomatic patients at high risk for coronary events, to improve the diagnostics of acute arterial disease events and to monitor the progression of atherosclerosis under treatment.

Atherosclerosis is a systemic and frequently pan-arterial disease. Histopathological studies [10], [11] have unravelled strong correlations between morphological and inflammatory indices as well as lipid content between different arteries within an individual person. Moreover, prospective studies demonstrated that the plaque load or a previous vascular event in one vascular bed, for example in the carotid artery, indicates a strongly increased risk for the incidence of clinical events in another vascular bed, for example the coronary arteries [11]. In this study therefore, we, like others [10], [12], [13], [14], used relatively easily accessible atherosclerotic plaques for the proteomic search of biomarkers that are intended to be used for risk prediction, diagnostics and monitoring of atherosclerotic vascular diseases in other arteries or even in general. Specifically, we combined subtractive antibody phage display [15], [16] with mass spectrometry (MS) to identify proteins released from cultured atherosclerotic lesions into so-called secretomes [17]. In initial verification studies, several isoforms of one identified protein, namely junction plakoglobin (JUP), were found to be expressed and released by endarterectomized plaques and macrophages, and to be enriched in coronary thrombi as well as in plasma samples of ACS and CAD patients.

## Materials and Methods

Detailed methodological information is given in the on-line supplementary information.

### Ethics Statement

The use of the tissue material and plasma samples investigated in this study was approved by the Cantonal Ethical Committees in Basel and Zurich, respectively. All studies on human materials were performed in accordance with the 1964 declaration of Helsinki. Informed and written consent was obtained from the donating patients.

### Preparation of Secretome

**Figure 1:**
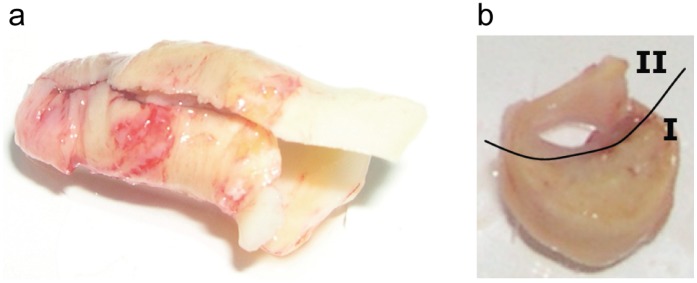
Thrombendarterectomy tissue for secretome production and immunohistochemistry. a) Tissue sample as it was received from the surgical department. b) The tissue was separated into the plaque part (I) for production of the plaque secretome, and the best preserved part (II) for production of the control secretome.

Secretomes were prepared from thrombendarterectomy specimens of carotid or iliac arteries [14] (Figure 1). In short, plaque and control tissues were incubated separately in protein-free medium for 24 hours. Subsequently, the media containing the secreted proteins, the so-called secretomes, were collected and used as antigens in subtractive antibody phage display.

### Selection of Antibodies

Secretomes were biotinylated with EZ-Link Sulfo-NHS-LC-Biotin. A large synthetic human phage-displayed single chain variable fragment (scFv) library, ETH-2-Gold [2], containing 3 billion individual antibody clones, was amplified as described [18]. Prior to the selections, the library was depleted of clones binding to some of the most common plasma proteins, including apo-transferrin, holo-transferrin, immunoglobulin G, fibrinogen and complement component 3.

**Figure 2:**
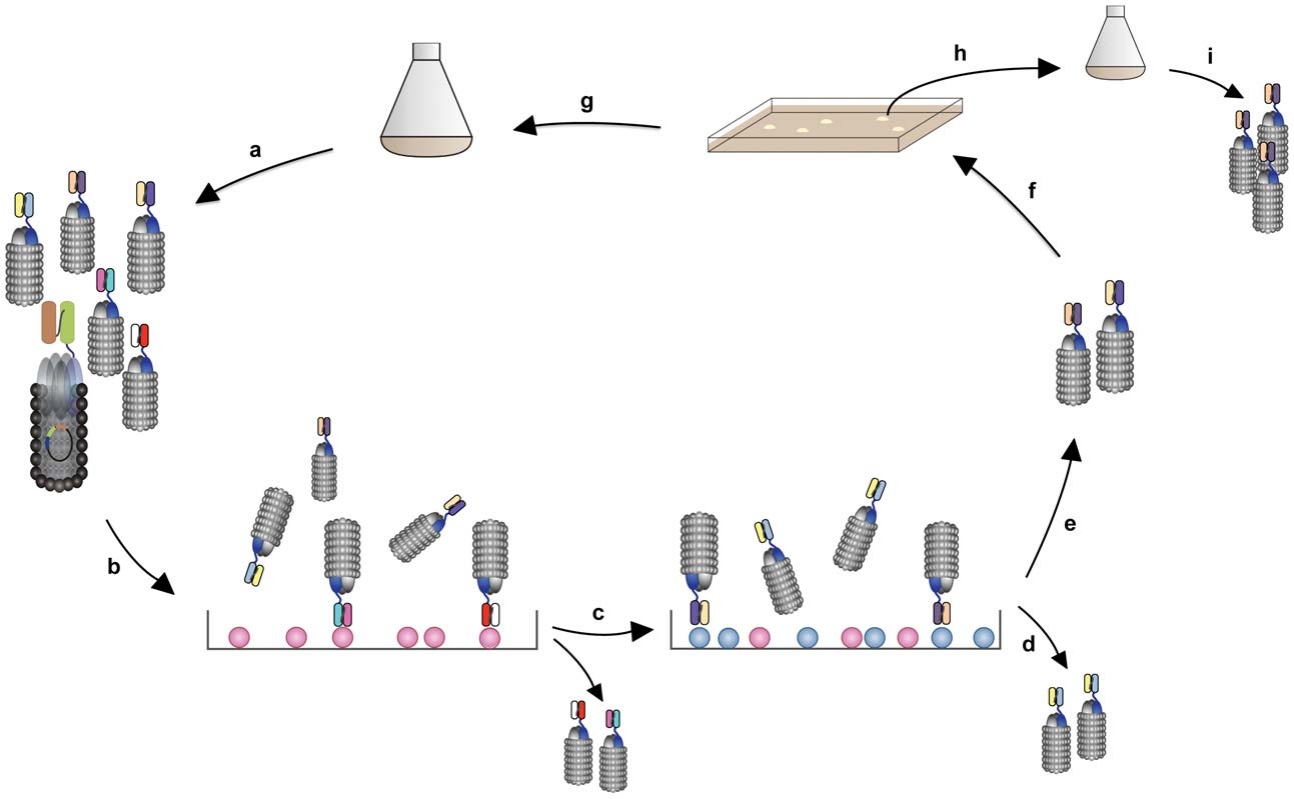
Schematic presentation of the selection strategy. a) The phage library, containing billions of different phages, was amplified from the bacterial stock. ScFv are fused to minor coat protein III and as such displayed on the phage surface. b) In a subtraction step, the phage library was incubated with secretome from the healthy control tissue. Binders to common proteins were removed in this way. c) Phages that did not bind to the control secretome were incubated with the atherosclerotic secretome in this panning step. d) Unbound phages were washed off, and e) bound phages were eluted and used to infect a suitable *E.coli* strain (*E.coli* TG1). f) Bacteria infected with the selected and eluted phages were plated on large agar plates. g) To further enrich for phages that specifically bind to the atherosclerotic secretome, the selection round was repeated. h) Single colonies were induced to produce monoclonal phages. i) Monoclonal phages were analysed for their reactivity with atherosclerotic versus control secretomes in ELISA. In total, six different selections were performed. For each separate selection the control and atherosclerotic secretomes from one individual patient were used. In order not to loose diversity, only two subtractive panning rounds were performed for each selection. To analyse whether enrichment of atherosclerotic secretome-specific binders had taken place, polyclonal phage pools (as obtained in step a) after each subtractive panning round (and of the unselected library as a control) were analysed in ELISA for reactivity with atherosclerotic and control secretomes.

Six selections (Figure 2) were done with two subtractive selection rounds each. Each selection was performed with an atherosclerotic and control secretome from a different patient. Subtraction was achieved by first removing phages binding to control secretome (i.e. secretome produced with the best preserved part of the tissue). The remaining, unbound phages were subsequently panned against the biotinylated plaque secretome from the same patient. Bound phages were captured using magnetic streptavidin-coated beads and eluted. *E.coli* TG1 was infected with the eluted phages and the bacteria were plated on large 2×TY agar plates containing ampicillin and 2% glucose. Phages were amplified from *E.coli* TG1 to be used in a second round. Two subtractive panning rounds were performed per selection. Polyclonal phage pools of each round were screened in an enzyme-linked immunosorbent assay (ELISA) for reactivity with control and plaque secretome.

### Screening of Single Clones

More than 500 single colonies were picked to produce monoclonal phages and the selected phages were tested for their ability to differentiate between plaque and control secretomes in ELISA. In addition, expression levels of soluble scFv were analysed by dot blotting and the cDNA encoding the scFvs of interest were sequenced.

### Production of Soluble scFv

The 34 clones showing the strongest difference in reactivity were cloned into a new vector and expressed as single chain variable fragment (scFv) with a 6×-His-tag and a VSV-G-tag, which replaced the original myc-tag. Soluble scFvs were induced with IPTG and isolated from the *E.coli* periplasm by sucrose extraction [19].

### ELISA

Reactivity of polyclonal phages, monoclonal phages and scFvs were analysed by ELISA. Antigens were coated on 96-well Maxisorb microtiter plates (Nunc) at a concentration of 0.1 µg/well in 100 µl of 50 mM carbonate buffer pH 9.6, overnight at 4°C. Plates were blocked with 400 µl MPBS containing 0.05% Tween 20 (MPBST) per well at RT for 2 hours. Phages and scFvs were diluted in MPBST and 100 µl were added to the plate and incubated at RT for 1 hour. The plates were washed eight times with PBST. To detect bound phages, HRP-conjugated anti-M13 monoclonal antibodies (mAB) (Amersham Biosciences) were added at a 5′000-fold dilution in MPBST and incubated at RT for one hour. To detect bound scFvs, anti-VSV-G or anti-myc antibodies were added (2′000-fold dilution), followed by incubation with HRP-labelled rabbit anti-mouse antibodies, 2′000-fold diluted. Bound HRP-conjugated antibodies were detected by 3,3′,5,5′-tetramethylbenzidine (TMB) conversion (Rockland Inc.). Reactions were stopped with 1 M H_2_SO_4_, and the absorbance at 450 nm was measured.

### Immunoprecipitation

Nine scFv antibodies with the strongest specificity for proteins in the plaque secretomes were used as baits for immunoprecipitation of their antigens in plaque and control secretomes. Proteins were precipitated from mixtures of two plaque and control secretomes, using magnetic Dynabeads for His-tagged proteins (Invitrogen) that captured the His-tagged scFvs. For information on unspecifically bound proteins, two control experiments were done. The precipitation was once done with magnetic Dynabeads without any antibody and once using unspecific scFv antibodies against TWIST1. TWIST1 acts as a transcriptional regulator and inhibits myogenesis [20] and has no known relationship with atherosclerosis or heart disease. Immunoprecipitated proteins were subsequently analysed by mass spectrometry.

### Protein Identification by Mass Spectrometry

All MS experiments were done at the Functional Genomics Center Zurich (FGCZ), a service centre of the University of Zurich. Immunoprecipitated proteins were concentrated using a speedvac system and dissolved in Laemmli buffer. Subsequently, proteins were separated by SDS-PAGE on Novex 12% Tris-Glycine 1 mm gels (Invitrogen) and stained with RotiBlue (Roth). Each lane was cut into 12 segments and put into individual wells of 96-well plates. All further sample work-up was executed on a liquid handling robot (TECAN). All gel pieces were subjected to reduction of disulfide bridges with DTT, alkylation of cysteines using iodoacetamide and in-gel trypsin digestion. The samples were then concentrated in a speedvac system and purified using ZipTip-µC18 (Milipore). Afterwards, the samples were pooled, concentrated and stored dry at −20°C until MS analysis. Samples were analyzed on an LTQ-Orbitrap XL mass spectrometer (Thermo Fischer Scientific, Bremen, Germany) coupled to an Eksigent-Nano-HPLC system (Eksigent Technologies, Dublin (CA), USA). The obtained data were searched against a human-contaminant database (human database including usual protein contaminants) using Mascot Server 2.2.

### Plasma Samples and Thrombi

Male subjects aged 54 to 65 admitted to the cardiac catheterisation laboratory at the University Hospital Zurich between December 2009 and October 2010 for coronary angiography either for suspected coronary artery disease, ACS, follow-up, or preoperatively before valve replacement or bypass surgery, were included in the study [21]. Patient characteristics are presented in Table 1. Four patients with peripheral artery occlusion disease (PAOD) were seen at the Department of Angiology at the University Hospital Zurich to undergo angiography. All four patients had undetectable plasma concentrations of (high-sensitive) cardiac troponin T. Thrombi were aspirated from coronary arteries of three ACS patients during angiography.

**Table 1:** Patient characteristics.

	Group		
	Control (n = 13)	CAD (n = 15)	ACS (n = 11)
Age (y)	59.1+/−4.3	59.9+/−3.9	59.2+/−4.9
BMI (kg/m2)	27.1+/−9.4	28.2+/−14	25.3+/−24
Blood pressure systolic (mmHg)	132+/−10.2	133+/−7.8	134+/−17
Blood pressure diastolic (mmHg)	80+/−4.2	79+/−4.3	84+/−9.7
Total cholesterol (mmol/l)	4.33+/−1.2	4.32+/−0.85	5.02+/−1.1
LDL cholesterol (mmol/l)	2.55+/−1.10	2.54+/−0.79	3.21+/−0.85
HDL cholesterol (mmol/l)	1.30+/−0.40	1.16+/−0.23	1.14+/−0.25
Triglycerides (mmol/l)	1.07+/−0.60	1.36+/−0.65	1.47+/−1.10
C-reactive protein (mg/ml)	1.33+/−1.03#	2.45+/−3.6#	9.45+/−10.3† ‡
Creatinine (µmol/l)	85+/−9.8	93+/−13.5	96+/−32

# p<0.05 compared to ACS group.

† p<0.05 compared to control group.

‡ p<0.05 compared to CAD group.

BMI = body mass index, LDL = low density lipoprotein, HDL = high density lipoprotein.

### Detection and Quantification of JUP and Other Candidate Proteins by Immunoblotting

Albumin and immunoglobulins were removed from human plasma samples with depletion columns (Qproteome columns, Invitrogen). Thrombi were fragmented and directly dissolved in SDS-PAGE loading buffer. After SDS-PAGE and Western blotting, JUP was detected with scFv antibodies 25G5 via its VSV-G-tag, or with anti-JUP monoclonal antibodies (mAb) 2C9, which was later replaced by mAb 2G9, from Lifespan Biosciences (Seattle, USA). As a positive control, GST-tagged recombinant JUP from Abnova (Taipei City, Taiwan) was used. Intensities of anti-JUP-immunoreactive bands were semi-quantitatively measured with ImageJ (National Institutes of Health [22]). To compare signals of different Western blots, one reference plasma sample was run on each gel for standardization. The intensities of all JUP bands were correlated to the intensity of the anti-JUP-immunoreactive band of this sample.

For rough JUP isoforms mapping the following antibodies were used: polyclonal ab134558 from Abcam (Cambridge, England), monoclonal LS-C21269, polyclonal LS-C118399, polyclonal LS-C77817, polyclonal LS-B1216 and monoclonal LS-B4364 (2G9) from Lifespan Biosciences (Seattle, USA), polyclonal CP2971 and monoclonal CM1111 from ECM Biosciences (Versailles, USA), monoclonal 610253 from BD Biosciences (Franklin Lakes, USA), and monoclonal P8087 from Sigma Aldrich (Saint Louis, USA).

Serpin B3 and plexin domain-containing protein 2 (Plxdc2) were detected by using commercial mAbs ab55733 from Abcam (Cambridge, England) and 4G10 from Abnova (Taipei City, Taiwan), respectively. Serpin B3 was also detected using the newly generated VSV-G-tagged scFv antibodies 36A8. As positive controls, GST-tagged recombinant serpin B3 and Plxdc2 from Abnova (Taipei City, Taiwan) were used.

### Immunohistochemistry

Tissue sections of coronary thrombi aspired from culprit coronary arteries of two ACS patients as well as endarterectomized atherosclerotic plaques were analyzed with the anti-JUP mAb 2C9, anti-serpin B3 mAb ab55733, anti-Plxdc2 mAb 4G10 and anti-CD68 antibodies (Clone PG-M1).

### Monocyte to Macrophage Differentiation

Peripheral blood monocytes were isolated from buffy coats (Blutspendedienst Zurich, Switzerland) by separation over Histopaque and subsequent isolation with magnetic CD14 beads [23]. Isolated cells were resuspended in RPMI-1640 medium with 5% fetal calf serum and 5% human serum (approximately 10^6^ cells/ml) and incubated in cell culture dishes. Cells were harvested at days 2, 5, 7 and 9 and lysed in lysis buffer (50 mM HEPES pH 8.0, 1 mM EDTA and 0.2% Triton X-100). GAPDH was detected with a mAb from Abnova.

Human acute monocytic leukemia cells (THP1, DSMZ, Germany) were plated in RPMI 1640 medium (Sigma) supplemented with 2 mM L-glutamine, 10% fetal bovine serum, 6.8 mM glucose, 1 mM sodium pyruvate and penicillin (100 U/mL)/streptomycin (100 ug/mL) in petri dishes. Next, cells were incubated with 200 nM phorbol-12-myristate-13-acetate (PMA) at 37°C and 5% CO_2_ for 1, 2, 3 and 4 days [23]. After the differentiation period, non-adherent cells were removed and remaining cells were harvested and lysed in ice-cold buffer containing 50 mM TRIS (pH 7.4), 150 mM NaCl, 1 mM EDTA, 1% NP-40, 7.5% glycerol and complete protease inhibitor cocktail (Roche) for 30 minutes. Finally, protein concentrations in cell lysates were determined by Bradford protein assay. β-Actin was detected with a mAb purchased from Sigma-Aldrich (Saint Louis, USA).

### Artificial Myocardial Infarction by Ligation in Swine

In two swine that did not have any atherosclerosis, myocardial infarction was artificially introduced by ligation (i.e. myocardial infarction without the occurrence of plaque rupture). Plasma samples were taken before (t = 0), three hours (t = 3 h) and three days (t = 3 d) after ligation. Cardiac troponin T (TnT) increased after ligation (data not shown).

### Measurement of Other Blood Parameters

Plasma levels of high sensitive troponin T, NT-proBNP (Roche E170), and CRP were measured by using the COBAS8000 autoanalyser and immunoassays from Roche diagnostics (Rotkreuz, Switzerland). Plasma concentrations of the endothelial markers sVCAM-1 and soluble E-selectin were measured by ELISAs from R&D Biosystems (Minneapolis, USA) following the instructions of the manufacturer. Plasma concentrations of squamous cells carcinoma antigen (SCC) were measured by using an immunoassay on the Kryptor immunoassay analyser from BRAHMS (Berlin, Germany).

### Data Analysis and Statistics

Genevestigator ™ [24] was used to explore known mRNA expression of identified proteins in human tissues and cells. Patient characteristics are given as mean and standard deviation. Non-parametric statistical tests were used for testing of equality of the biomarkers in the three patient groups (Mann-Whitney and Kruskal-Wallis) and to calculate correlations between the biomarkers (Spearman-Rho test).

## Results

### Phage Display-assisted Identification of 22 Proteins in Plaque Secretromes

Secretomes were prepared by incubating atherosclerotic and control tissues in protein-free medium for 24 hours (Figure 1). Six separate selections were performed using six different pairs of plaque and control secretomes, obtained from six different patients, with two subtractive panning rounds in each selection (Figure 2). Subsequently, more than 500 single clones of antibodies expressing phages were analysed by ELISA. The genes of 40 scFvs, which showed the strongest immunoreactivity with independent sets of secretomes, were sequenced. Because of strong sequence similarities or absent scFvs gene expression, six clones were discarded. To allow immunodetection in plasma without interference by endogenous myc, cDNAs of the 34 remaining scFvs were re-cloned into vector pUC119 to replace myc-tags with VSV-G- and 6×-His-tags. After verification of the successful insertion scFvs were produced in *E.coli* TG1 production strain and isolated from the periplasmic fractions. In subsequent ELISAs, nine of the 34 scFvs were found to react stronger with the plaque secretomes than with the respective control secretomes from at least three of four patients. They were, therefore, chosen for immunoprecipitation of their antigens both in pooled control and in pooled plaque secretomes. Two control experiments were performed by using beads with either no or inert antibodies directed against TWIST1. MS analysis identified 105 proteins that were specifically immunoprecipitated by one of the scFvs in either the plaque or the control secretome or both, but not by the inert anti-TWIST1-antibodies or the antibody-free beads. 22 proteins (Table 2) were identified in this affinity-enrichment approach, but not with direct MS analysis of the secretomes. Genevestigator, a database containing mRNA expression data, was used to explore the expression of these 22 proteins in human cells and tissues. In this way, some proteins could be excluded from further investigation, as e.g. alpha-2-macroglobulin due to its high expression in liver. Primary investigations, also because of the availability of commercial antibodies, were focused on three out of the 22 proteins, namely junction plakoglobin (JUP), serpin B3, and plexin domain-containing protein 2 (Plxdc2).

**Table 2:** Overview of the 22 proteins identified by subtractive phage display combined with mass spectrometry.

Protein	Function	Accession number	Nr of unique peptides	Protein coverage (%)
26S proteasome non-ATPase regulatory subunit 9	Chaperone during 26S proteasome assembly	O00233	2	8.5
Alpha-2-macroglobulin	Protease inhibitor	P01023	3	2.2
Annexin A5	Coagulation inhibitor	P08758	2	5.3
Caspase-14	Non-apoptotic caspase involved in epidermaldifferentiation	P31944	2	8.7
cDNA FLJ60424, highly similar to junction plakoglobin	Unknown	B4DE59	4 *	13
cDNA FLJ78440, highly similar to human lactoferrin	Unknown	A8K494	13	12
Collagen alpha-1(I) chain	Structural protein; member of group I collagens	P02452	17	12
Collagen alpha-1(III) chain	Structural protein; member of group III collagens	P02461	2	1.6
Desmocollin-1	Structural component of intercellular desmosomejunctions	Q08554	4	4.9
Extracellular glycoprotein lacritin	Modulates secretion by lacrimal acinar cells	Q9GZZ8	2	17
Filaggrin-2	Regulation of epithelial homeostasis	Q5D862	2	1.0
Glyceraldehyde-3-phosphate dehydrogenase	Glycolysis; Trigger of apoptosis	P04406	3	9.0
Junction plakoglobin	Structural protein of desmosomes and intermediate junctions	P14923	4	8.1
Keratin type I, cytoskeletal 17	Belongs to the intermediate filament family	Q04695	7	26
Keratin type I, cytoskeletal 19	Belongs to the intermediate filament family; involved in organization of myofibers	P08727	6	32
Keratin type I, cytoskeletal 78	Belongs to the intermediate filament family	Q8N1N4	4	9.6
Keratin type I, cytoskeletal 80	Belongs to the intermediate filament family	Q6KB66	3	8.2
Lipocalin-1	Belongs to lipocalin family (lipophile binders)	P31025	4	21
Mucin-5AC	Gel-forming glycoprotein of gastric and respiratory tract epithelia	P98088	2	0.6
Nuclear receptor coactivator 1	Coactivator of different nuclear receptors	Q15788	3	1.7
Plexin domain-containing protein 2	May play of role in tumor angiogenesis	Q6UX71	4	6.6
Serpin B3	Protease inhibitor	P29508	2	4.9

* 2 peptides are shared with junction plakoglobin and 2 peptides are shared with keratin type I, cytoskeletal 19.

### Serpin B3 and Plxdc2

Immunohistochemistry clearly demonstrated the presence of serpin B3 in endarterectomised plaques (Figure S1a). A protein band of 42 kD, corresponding to the molecular weight of serpin B3, could be detected in plaque secretome, but not control secretome, with a commercial mAb against serpin B3, although also other protein bands were detected (Figures S1b and S1c). Plasma analysis of serpin B3 by an immunoassay for squamous cell carcinoma antigen (SCC), which detects both serpin B3 (SCCA1) and serpin B4 (SCCA2), did not unravel any significantly different concentrations between controls (0.45±0.27 ng/L, n = 15), ACS (0.60±0.54 ng/L, n = 13) and stable CAD patients (0.57±0.29 ng/L, n = 15).

Plxdc2 was detected in endarterectomised plaque tissue as well, although the immunohistochemical staining was weaker than that of serpin B3 (Figures S2a). In coronary thrombi, Plxdc2 was identified in cellular but not in extracellular parts (Figure S2b). Immunoblotting using a commercial mAb against Plxdc2 did not detect this antigen in plasma samples from patients and healthy controls (Figure S2c).

### JUP in Atherosclerotic Plaques and their Secretomes

**Figure 3:**
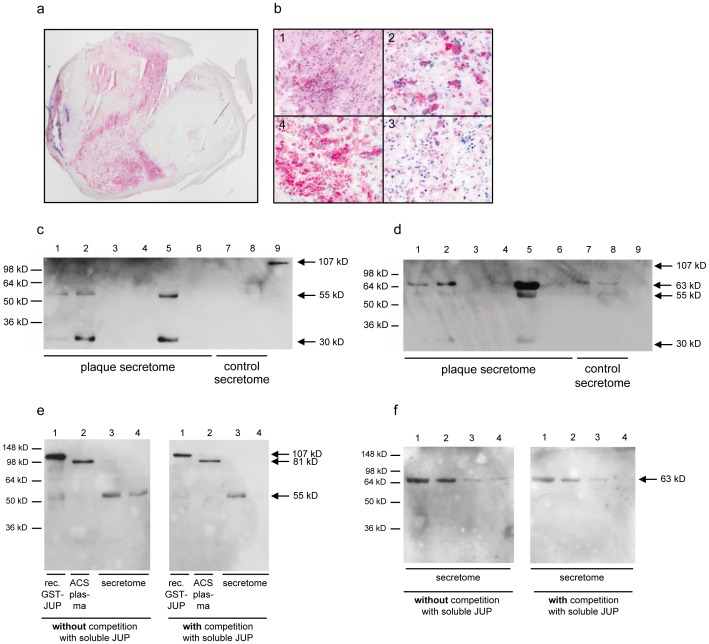
Detection of JUP in plaques by immunohistochemistry and in secretome by immunoblotting. a) Overview of JUP immunoreactivity on endarterectomised tissue. Strong staining in the atherosclerotic plaque tissue is observed. b) Clockwise from top left (400-fold magnification): H&E staining (1), anti-CD68 (2), negative control (3), and anti-JUP staining (4). c and d) Six plaque secretomes (lanes 1–6), two control secretomes (lanes 7 and 8) and GST-tagged JUP (lane 9, 107 kD) were immunoblotted with anti-JUP mAb 2C9 (c) and scFv 25G5 (d). e) Competition experiment with mAb 2G9 (which replaced 2C9). Western blots containing recombinant GST-tagged JUP (lane 1, 107 kD), ACS plasma (lane 2), and secretome (4.5 µl in lane 3 and 1.9 µl in lane 4) were incubated with mAb 2G9, which was (blot on the right) or was not (blot on the left) pre-incubated with soluble, recombinant GST-tagged JUP protein. f) Competition experiment with scFv 25G5. Western blots containing different amount of atherosclerotic plaque secretome (lane 1∶4.5 µl, lane 2∶1.9 µl, lane 3∶0.9 µl, lane 4∶0.4 µl) were incubated with scFv 25G5, which was (blot on the right) or was not (blot on the left) pre-incubated with soluble, recombinant GST-tagged JUP protein. For all immunoblots: known molecular weights of protein markers are depicted on the left and estimated molecular weights of detected protein bands are depicted on the right of both Western blots.

Immunohistochemical analysis of endarterectomy specimens of carotid arteries with the commercially available anti-JUP antibodies mAb 2C9 showed strong immunoreactivity with dispersed cells in the intima (Figure 3a). By their morphological appearance and additional immunoreactivity with specific anti-CD68 antibodies, these JUP-positive cells correspond to macrophages (Figures 3b).

The monoclonal mAb 2C9 (and its replacement 2G9) antibodies did not identify JUP in plaque secretomes with its expected size of 81 kD (JUP-81) (Figure 3c), but reacted strongly with two proteins with apparent molecular masses of 30 kD (JUP-30) and 55 kD (JUP-55), which may represent degradation products, alternative splice variants, or homologues of JUP. Both proteins were detected in atherosclerotic secretomes but not in control secretomes. Similar results were obtained with other antibodies against JUP (data not shown). The two bands of 30 kD and 55 kD were also detected in the same secretomes by scFv 25G5 (Figure 3d), the scFv by which JUP was initially identified. ScFv 25G5 additionally reacted with a protein of approximately 63 kD (JUP-63), which was not detected by the commercial antibodies 2C9. We hypothesize that the 63 kD protein represents a protein which is encoded by cDNA FLJ60424 (Swissprot ID B4DE59), because it is highly similar to JUP, has a predicted molecular weight of 63 kD and because it was also identified by MS as one of the antigens immunoprecipitated with scFv 25G5. In addition, binding of the antibodies 2C9/2G9 and 25G5 to JUP-81, JUP-63 and JUP-55 on Western blots could be reduced by competition with soluble recombinant JUP (Figures 3e and 3f), further strengthening the hypothesis that these bands are indeed JUP isoforms. The 745 and 563 amino acids encompassing sequences of JUP and cDNA FLJ60424, respectively, share an identical N-terminal sequence of 303 amino acids. MS identified four peptides that can be assigned to both intact JUP and cDNA FLJ60424. In addition, two and four peptides were identified that were assigned to JUP and cDNA FLJ60424, respectively (Figure S3).

### Increased Plasma Levels of JUP Isoforms in Patients with Coronary Artery Disease and Peripheral Arterial Occlusion Disease

**Figure 4:**
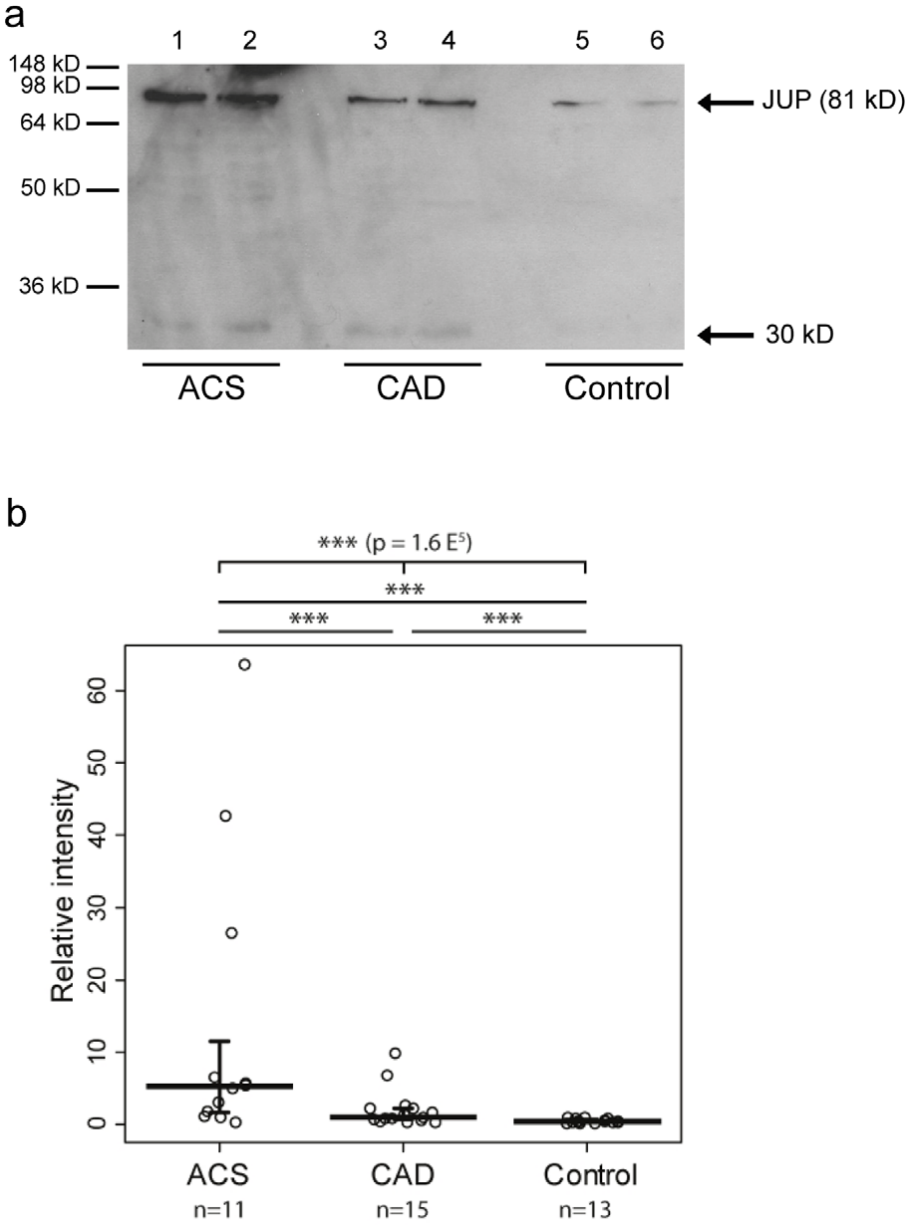
JUP plasma levels in patients with ACS, CAD and controls. a) Detection of JUP-81 by immunoblotting with mAb 2C9. b) Semi-quantitative analysis of Western blots. JUP plasma levels were correlated to JUP in a reference plasma that was run on each blot.

As depicted in Figure 4a, immunoblotting with the commercial anti-JUP mAb 2C9 detected the 81 kD band of intact JUP in plasma samples, which were depleted of albumin and IgG. The only available commercial ELISA for JUP was found to be not sensitive enough for detection of JUP in plasma samples. A semi-quantitative immunoblotting approach revealed that median JUP plasma levels are 2.4- and even 14.1-fold elevated in the plasma of 15 patients with stable CAD patients (p<0.01) and 11 patients with ACS (p<0.01), respectively, as compared to 13 CAD-free controls (Figure 4b). The three patients with very high JUP levels (27-, 34-, and 64-fold higher than the median level in control plasmas) did not differ from the other ACS patients by their clinical presentation or troponin T levels (0.34, 0.54 and 4.5 µg/l, respectively, compared to the range of 0.01 to 8.0 µg/l). After removal of these three patients from the statistical analysis, JUP-81 was present in ACS plasmas at 10.6-fold higher median concentration than in control plasma (p<0.01).

Plasma levels of JUP-81 correlated with plasma concentrations of troponin T (r = 0.44, p<0.01), NT-proBNP (r = 0.41, p<0.01) and CRP (r = 0.51, p<0.01). Correlations with plasma levels of the soluble endothelial activation marker sVCAM-1 just missed the level of significance (r = 0.31, p = 0.094). JUP concentrations did not correlate with E-selectin plasma levels.

**Figure 5:**
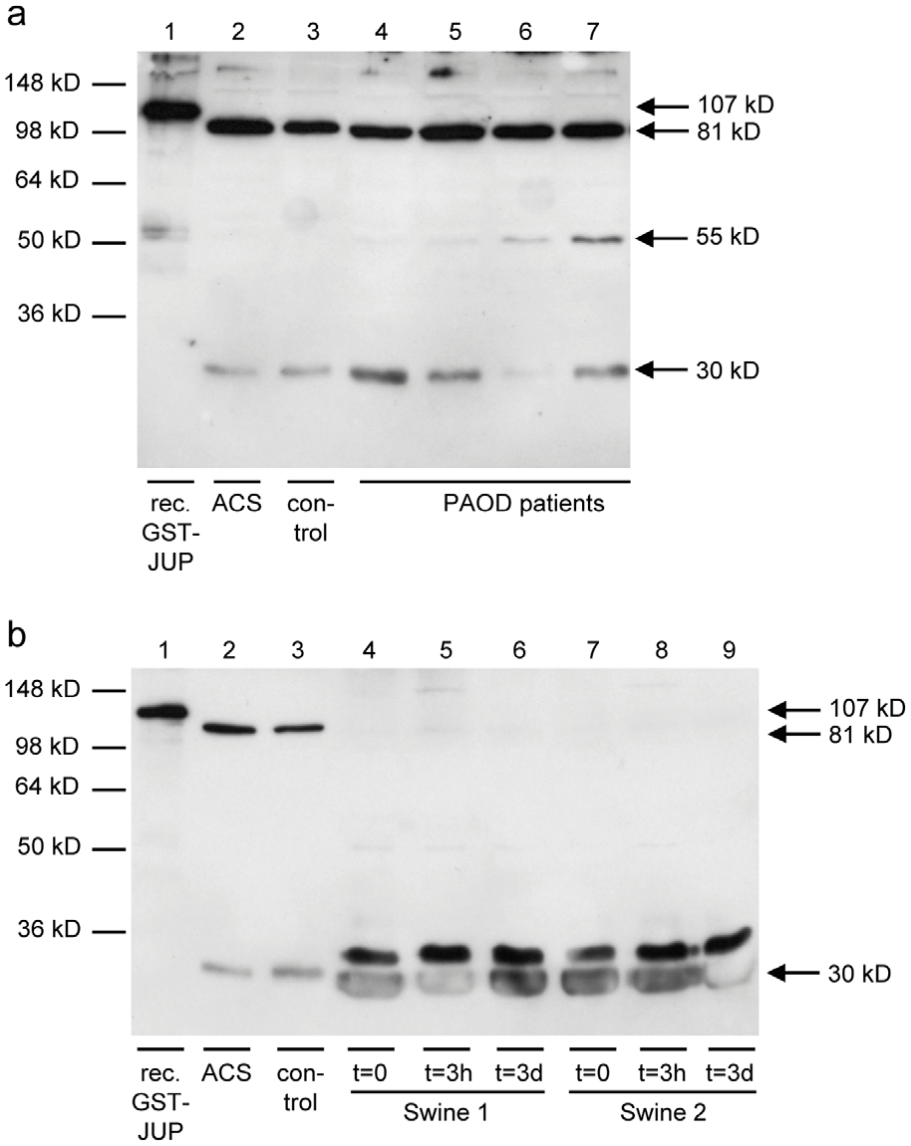
Detection of JUP isoforms in plasma from PAOD patients with atherosclerosis and in plasma from a swine model of myocardial infarction without atherosclerosis and plaque rupture. a) Western blot containing recombinant GST-tagged JUP (lane 1, 107 kD), ACS plasma (lane 2), control plasma (lane 3) and plasma from four PAOD patients (lanes 4 to 7) were detected with mAb 2G9 (which replaced 2C9). JUP-55 and JUP-30 are clearly detected besides JUP-81. b) Western blot containing recombinant GST-tagged JUP (lane 1, 107 kD), ACS plasma (lane 2), control plasma (lane 3) and plasma samples from swine before ligation (lanes 4 and 7), three hours after ligation (lanes 5 and 8) and three days after ligation (lanes 6 and 9) were detected with mAb 2G9. JUP-81 was not detected in the swine samples, whereas JUP-30 and a protein band with a slightly larger molecular weight were detected with similar intensities before and after ligation.

The presence of JUP isoforms in plasma from patients with PAOD was analysed in four patients (Figure 5a). Besides JUP-81, also JUP-55 and JUP-30 were detectable in these plasma samples.

To rule out the myocardial origin of JUP, we analysed plasma samples of swine, in which myocardial infarction was artificially introduced by ligation. Since swine JUP is highly homologous to human JUP (98.5% homology on the amino acid level), mAb 2G9 is highly likely to react with swine JUP as well and 2G9 was therefore applied to western blots containing samples of this model of atherosclerosis-free myocardial damage. We did not find detectable plasma levels of JUP-81, and JUP-30 was detected at similar levels before and after ligation (Figure 5b).

### JUP in Coronary Thrombi of ACS Patients

**Figure 6:**
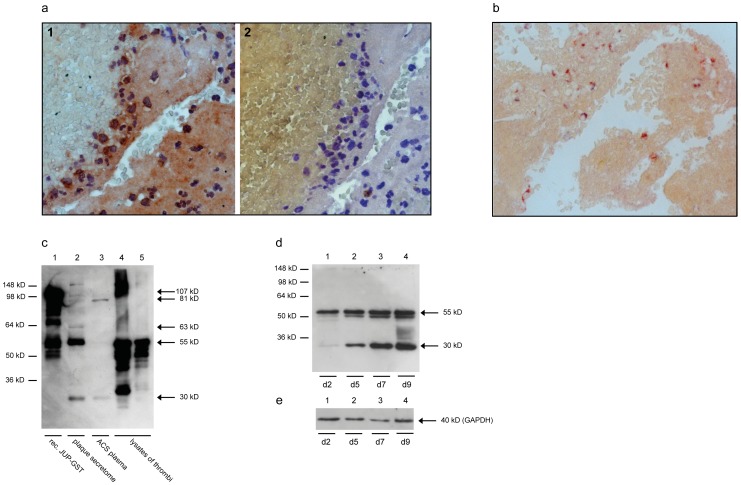
Detection of JUP in thrombi. a) Tissue section of a coronary thrombus stained with anti-JUP mAb 2C9 and labelled goat anti-mouse antibodies (brown) (1, on the left) or with the secondary labelled goat anti-mouse antibody only as a negative control (2, on the right). Nuclei are stained in blue. Immunoreactivity of JUP is detected intra- as well as extracellularly. b) Tissue section of a coronary thrombus, double-stained with anti-JUP mAb 2C9 (red) and the macrophage marker anti-CD68 mAb (black). In addition, an extracellular staining of the fibrin platelet clot was observed. c) Detection of JUP in lysates of coronary thrombi by immunoblotting. A Western blot with recombinant GST-tagged JUP (lane 1, 107 kD), plaque secretome (lane 2), plasma from an ACS patient (lane 3), and lysates of two thrombi (lanes 4 and 5) were immunoblotted with mAb 2C9. d and e) Western blot of macrophages, that differentiated from monocytes isolated from peripheral blood on day 2 (lane 1), day 5 (lane 2), day 7 (lane 3) and day 9 (lane 3), was immunoblotted with mAb 2G9 (which replaced 2C9) (d) and an anti-GAPDH mAb (e) as a loading control.

Thrombi isolated from coronary culprit lesions of two ACS patients were analyzed by immunohistochemistry, using the commercial mAb 2C9 for detection of JUP. As demonstrated in Figure 6a, JUP was clearly detected in cells, as well as extracellularly. By co-staining of JUP with an anti-CD68 mAb as a macrophage marker, it was shown that cells expressing CD68 clearly demonstrated JUP immunoreactivity as well (Figure 6b). To unravel the isoform-identity of these anti-JUP-immunoreactive proteins, two thrombi were dissolved in SDS-PAGE loading buffer, separated by SDS-PAGE and immunoblotted with anti-JUP mAb 2C9 (Figure 6c) and 25G5 (not shown). Neither the 81 kD nor the 63 kD protein bands could be detected. However, the two protein bands of 55 and 30 kD, which were also found in the secretomes, as well as several protein bands with estimated molecular masses of 40, 45, and 100 kD were clearly detected in the thrombus lysates.

### JUP Isoforms are Expressed by Macrophages *in vitro*

Peripheral blood monocytes were isolated from buffy coats of human blood donors and differentiated into macrophages by cell cultivation for 2, 5, 7 and 9 days. Expression of JUP isoforms was analysed by Western blotting. As shown in Figure 6d, the expression of both JUP-55 and JUP-30 isoforms increased time-dependently from day 2 to day 9.

**Figure 7:**
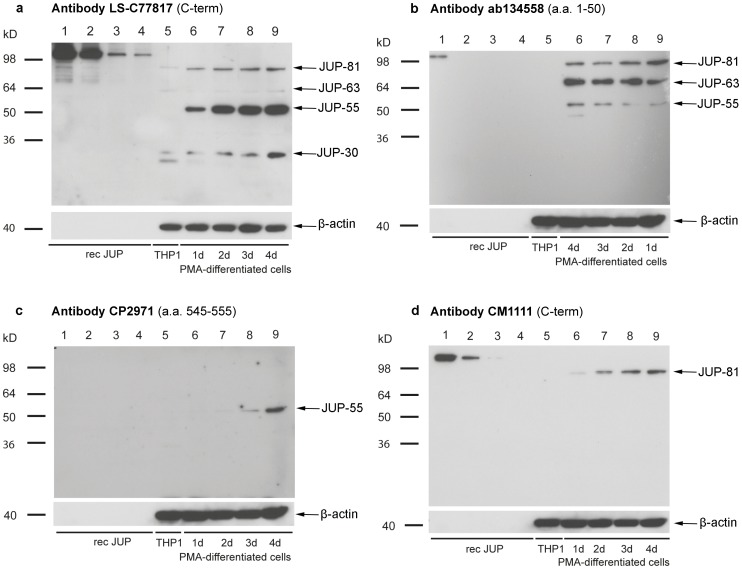
Detection of JUP isoforms in macrophage-like cells. To differentiate THP1 into macrophage-like cells, cells were stimulated by PMA for 1, 2, 3 and 4 days. JUP isoforms were analyzed by Western blotting using anti-JUP antibodies LS-C77817 (a), ab134558 (b), CP2971 (c) and CM1111 antibody (d). 200, 100, 50 and 25 ng of recombinant GST-tagged JUP was used as positive control (lanes 1–4, respectively). Lane 5 contains lysates of undifferentiated THP1 cells (monocytes) and lanes 6–9 contain lysates of differentiated THP1 cells (macrophages) after 1, 2, 3 and 4 days of differentiation (note opposite order of loading in panel b)). β-actin was employed as a loading control. CP2971 reacts only weakly with recombinant JUP on Western blots (no visible reaction detected here).

Similar results were seen in THP1 monocytes that were transformed into macrophages by treatment with phorbol esters (Figure 7). All four JUP isoforms, namely JUP-81, JUP-63, JUP-55 and JUP-30, were detectable in the macrophage state, but except JUP-30, none of them in the monocyte state (Figure 7a). Especially the expression of JUP-81 and JUP-55 increased with time of differentiation and cultivation.

### Immunomapping of JUP Isoforms

**Figure 8:**
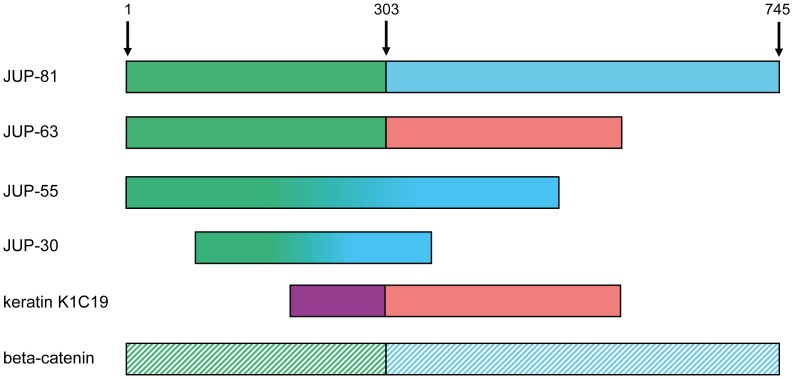
Schematic overview of the four different JUP isoforms. JUP-81 and JUP-63 have an identical N-terminus (the N-terminal 303 amino acids), and JUP-63 further shares its C-terminus with cytokeratin 19 (K1C19). The sequences of JUP-55 and JUP-30 are currently unknown but they contain epitopes that are shared with JUP-81 and are thought to be located in the N-terminal region of native JUP. JUP-30 lacks (at least) the N-terminal 50 amino acids of JUP-81. JUP-81, which is also referred to as gamma-catenin, is furthermore homologous to beta-catenin. Amino acids 1, 303 and 745 of JUP-81 are indicated with arrows.

Because of the clear signals, we used THP1-macrophages to localize the three atypical JUP isoforms relative to JUP-81 by the use of different anti-JUP antibodies with characterized epitopes (Table 3). As demonstrated in Figure 7a, antibody LS-C77817 (with its epitope located in the C-terminal region) reacted with all four isoforms, although more weakly with JUP-63 and JUP-30. Both JUP-63 and JUP-55 immunoreacted with several antibodies with epitopes in the N-terminal proportion of the molecule, including antibody ab134558 with an epitope within amino acid residues 1–50 (Figure 7b). Therefore, JUP-63 and JUP-55 appear to represent N-terminal parts of JUP-81. Antibody CP2971, with its epitope residing between amino acid residues 545 and 555 only reacted with JUP-55 but not JUP-63 or JUP-30 (Figure 7c). This is in agreement with the alignment of the first 303 amino acid residues of JUP-81 with JUP-63 (Figure S3). Also other antibodies with epitopes located at the C-terminus of JUP-81 did not react with JUP-63. Because of lacking reactivity of ab134558 (a.a 1–50) JUP-30 appears not to share the first 50 amino acids with the other JUP isoforms and may be located between amino acid residues 50 and 545. Antibody CM1111 (Figure 7d), which has a C-terminal epitope, does only react with JUP-81, again indicating that JUP-55 and JUP-30 are located in the N-terminal, rather than in the C-terminal, part of JUP-81. Figure 8 provides a schematic overview of the hypothetical positions of the different JUP isoforms.

**Table 3:** Reactivities of ten different commercial anti-JUP antibodies with the four JUP isoforms.

Antibody	Immunogen (epitope)	Rec. JUP	JUP-81	JUP-63	JUP-55	JUP-30
ab134558	a.a. 1–50	+	+	+	+	-
LS-C21269	full-length JUP (a.a. 45–114)	+	+	-	-	-
CP2971	a.a. 545–555	+	-	-	+	-
610253	a.a. 553–738	+	+	-	-	-
LS-C118399	C-terminal region	+	+	-	-	-
LS-C77817	C-terminal region	+	+	+/−	+	+
LS-B1216	C-terminal region	+	-	-	+	-
CM1111	C-terminal region	+	+	-	-	-
LS-B4364 (2G9)	full-length JUP	+	+	-	+	+
P8087	chicken JUP	+	+	+	+	-

a.a. = amino acids; Rec. JUP = recombinant, GST-tagged JUP.

## Discussion

By integrating subtractive phage display with MS we identified JUP and its smaller isoforms as potential biomarkers of atherosclerosis. In fact, we provide three lines of evidence for this conclusion. First, by using a hypothesis-free proteomics approach we identified JUP-81 and JUP-63, a protein encoded by the related cDNA FLJ60424, in secretomes of atherosclerotic plaques. The fact that these proteins are released from the endarterectomised tissue into the culture medium indicates that they might well be released *in vivo* into the blood stream as well, which is a prerequisite for any potential blood biomarker of atherosclerotic processes. In line with this initial discovery, atherosclerotic but not control secretomes contain proteins with apparent molecular weights of 30, 55, 63 and 81 kD that immunoreact with our discovering phage display antibody scFv 25G5, as well as independent commercial antibodies against JUP. Immunoreactivity of several antibodies including the scFv screening antibody with the JUP isoforms could be reduced by pre-incubation of the antibodies with JUP, indicating that these bands do represent JUP isoforms. Second, compared to healthy controls, median concentrations of JUP-81 were increased by factors higher than 2 and 14 in plasma of patients with stable CAD and ACS, respectively. Third, macrophages of endarterectomized plaques as well as monocytes, macrophages and fibrin platelet clots of coronary thrombi of ACS patients show a strong anti-JUP-immunoreactivity. Lysates of thrombi contained the same 55 kD JUP-antigen which was detected in atherosclerotic secretomes as well as cultivated monocyte-derived macrophages.

JUP is a protein component of desmosomes, which are junction complexes with essential structural functions in tissues that experience mechanical stress [25]. Desmosomes connect neighbouring cells through their transmembrane cadherins (desmocollin and desmoglein). The cytoplasmic tails of cadherins are connected through JUP, plakophilin and desmoplakin to the intermediate filaments. The essential physiological role of JUP for regular function of desmosomes in the myocardium is indicated by the findings of premature cardiac death of JUP knockout mice [26], [27] and arrhytmogenic right ventricular cardiomyopathy in patients carrying mutations in the JUP gene [28], [29]. Furthermore, the importance of JUP for endothelial integrity is indicated by the results of several *in vitro* experiments [30], [31]. Recent data by Sun *et al.* demonstrated that overexpression of the disintegrin and metalloproteinase 15 (ADAM15), a metalloprotease that was recently identified as a regulator of endothelial permeability, led to dissociation of gamma-catenins (JUP) from VE-cadherin [32]. At least in theory, the increased JUP plasma levels in ACS and CAD patients may hence be explained by either myocardial or endothelial damage or both. In agreement herewith, concentrations of JUP-81 in human plasma correlated with other biomarkers of myocardial damage (troponin T, r = 0.44) and dysfunction (NT-proBNP, r = 0.41) as well as endothelial dysfunction (sVCAM1, r = 0.31). In addition, we found that macrophages of endarterectomized plaques and coronary thrombi, as well as macrophages differentiated either from peripheral blood monocytes or THP1 monocytes *in vitro*, express JUP isoforms. This finding was unexpected, since macrophages are single cells that do not form continuous and adherent stretches of cells, which are interconnected by desmosomes or junctions. In addition, JUP isoforms could be detected in plasma from patients with PAOD (having peripheral atherosclerotic plaques without suffering a myocardial infarction), but not in a swine model in which non-atherosclerotic myocardial infarction was induced by ligation. Taken together, these findings indicate that JUP isoforms are produced by macrophages in the atherosclerotic plaques, thrombi and differentiated from monocytes *in vitro*, rather than being released from the myocardium.

In addition to JUP-81, a 63 kD JUP-homologue encoded by cDNA FLJ60424, was identified by both MS and Western blotting of atherosclerotic secretomes. Both the 81 kD- and 63 kD-proteins were immunoprecipitated from secretomes with scFv 25G5 and were recognized by several commercial antibodies with epitopes in the N-terminus of JUP-81. Furthermore, reactivity of scFv 25G5 with JUP-63 could be competed by pre-incubation of the antibody with recombinant JUP-81. This suggests that scFv 25G5 recognizes an epitope that is shared by JUP-81 and JUP-63 and is thus likely to be located in the common N-terminal part of both proteins. Currently, nothing is known about the 63 kD JUP isoform, since it has only been described as a coding DNA submitted into the databases of EMBL [33], GenBank [34] and DDBJ [35]. Cloning and expression of JUP-81 and JUP-63 in bacterial or mammalian cells are needed to gain more insight into the location of the epitope that is recognized by 25G5 and to generate antibodies and standards for sandwich ELISAs to quantify these proteins in patient samples. In addition, high-affinity antibodies that specifically recognize JUP-63 will be essential to characterize its tissue expression pattern and function.

It is not clear whether JUP-55 and JUP-30 are degradation products of JUP-81 and/or JUP-63, or if these two proteins represent additional, as yet unknown (alternative splicing) variants of JUP. Blast database searches with the JUP sequence identified highly identical, smaller sequences that could represent the 30 kD band, but no sequence was found that could correspond to a protein band of 55 kD. Mapping with commercial antibodies revealed that JUP-55 is located in the N-terminal part of JUP and that JUP-30 may be located somewhere between amino acid residues 50 and 545 (Figure 8). In further studies, JUP isoforms (fragments) will be identified by mass spectrometry to gain information about their sequences and origin.

In conclusion, this is the first report of JUP and its isoforms in the context of atherosclerosis and cardiovascular disease. The increased JUP concentrations in plasma of patients with stable CAD and ACS suggest JUP and its isoforms as potential biomarkers for atherosclerosis. The development of quantitative, high-throughput immunoassays for JUP and for the JUP isoforms, as well as their subsequent application in different clinical studies are needed to validate JUP as a biomarker for diagnosis, prognosis or monitoring of atherosclerosis.

## Supporting Information

Figure S1 Detection of serpin B3 by immunohistochemistry and immunoblotting.
(DOC)

Figure S2 Detection of Plxdc2 in endartectomised tissue and coronary thrombi by immunohistochemistry, and in plasma samples by immunoblotting.
(DOC)

Figure S3 Alignment of the amino acid sequences of JUP and of the JUP variant encoded by cDNA FLJ60424.
(DOC)

Methods S1 Provides detailed methodological information.
(DOC)
