# Supplementary material for: Antibody Phage Display Assisted Identification of Junction Plakoglobin as a Potential Biomarker for Atherosclerosis
Source: PLoS One. 2012 Oct 24;7(10):e47985. doi: 10.1371/journal.pone.0047985 (PMC3480477; doi:10.1371/journal.pone.0047985)
Supplement: Figure S1 — Detection of serpin B3 by immunohistochemistry and immunoblotting. (DOC) [file pone.0047985.s001.doc]

**
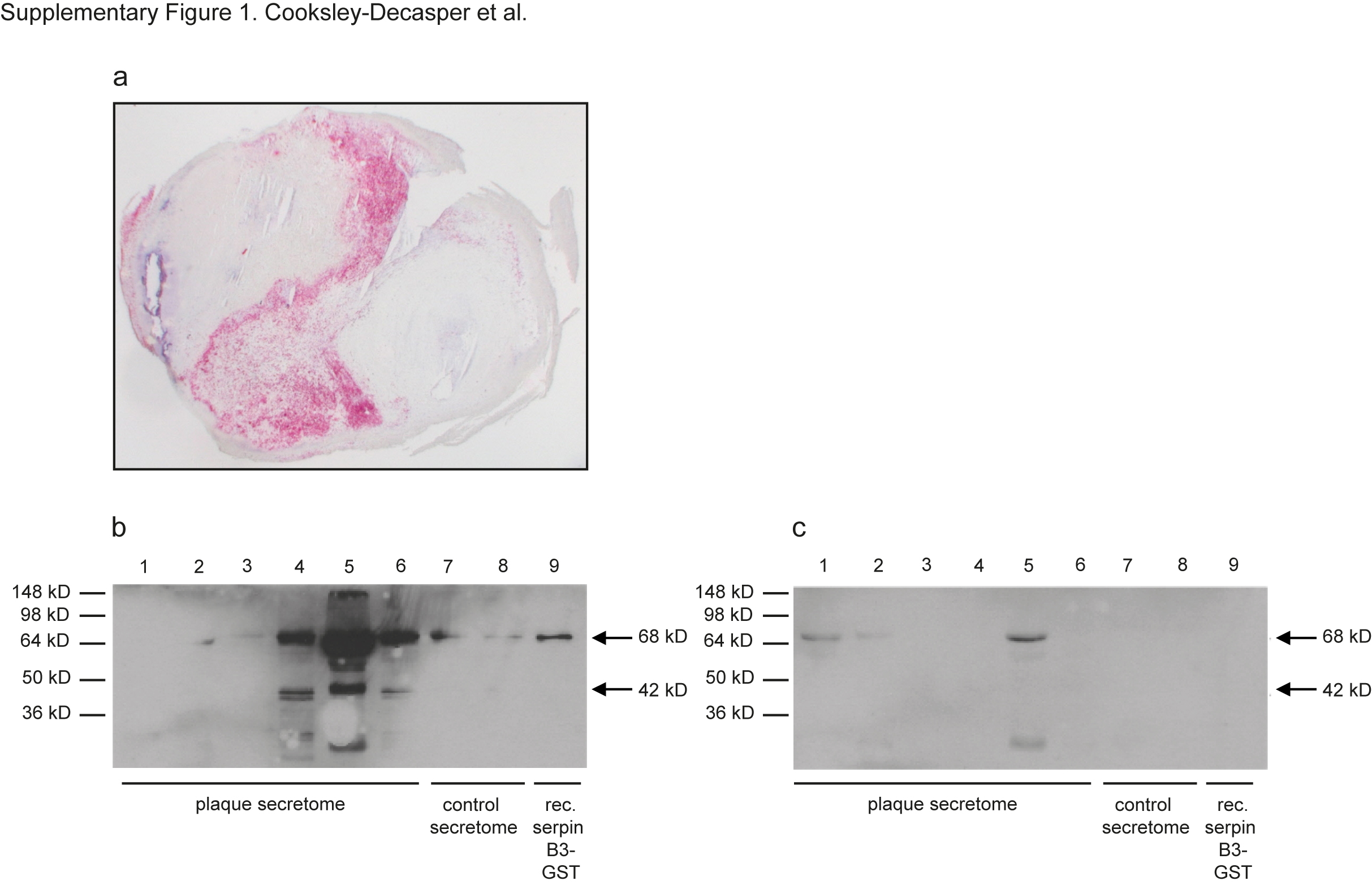
**

**Supplementary Figure S1. Detection of serpin B3 by immunohistochemistry and immunoblotting.** a) Strong immunohistochemical detection of serpin B3 in the atherosclerotic part of endarterectomised tissue. b and c) Proteins from six plaque secretomes (lanes 1 - 6), from two control secretomes (lanes 7 and 8), as well as recombinant, GST-tagged serpin B3 (lane 9, 68 kD) were separated on 12.5% polyacrylamide gels and transferred to nitrocellulose membranes for detection with commercial and scFv antibodies. b) Reactivity of the commercial anti-serpin B3 antibodies. c) Reactivity of the affinity-selected scFv antibodies 36A8.
