# Supplementary material for: Antibody Phage Display Assisted Identification of Junction Plakoglobin as a Potential Biomarker for Atherosclerosis
Source: PLoS One. 2012 Oct 24;7(10):e47985. doi: 10.1371/journal.pone.0047985 (PMC3480477; doi:10.1371/journal.pone.0047985)
Supplement: Figure S2 — Detection of Plxdc2 in endartectomised tissue and coronary thrombi by immunohistochemistry, and in plasma samples by immunoblotting. (DOC) [file pone.0047985.s002.doc]

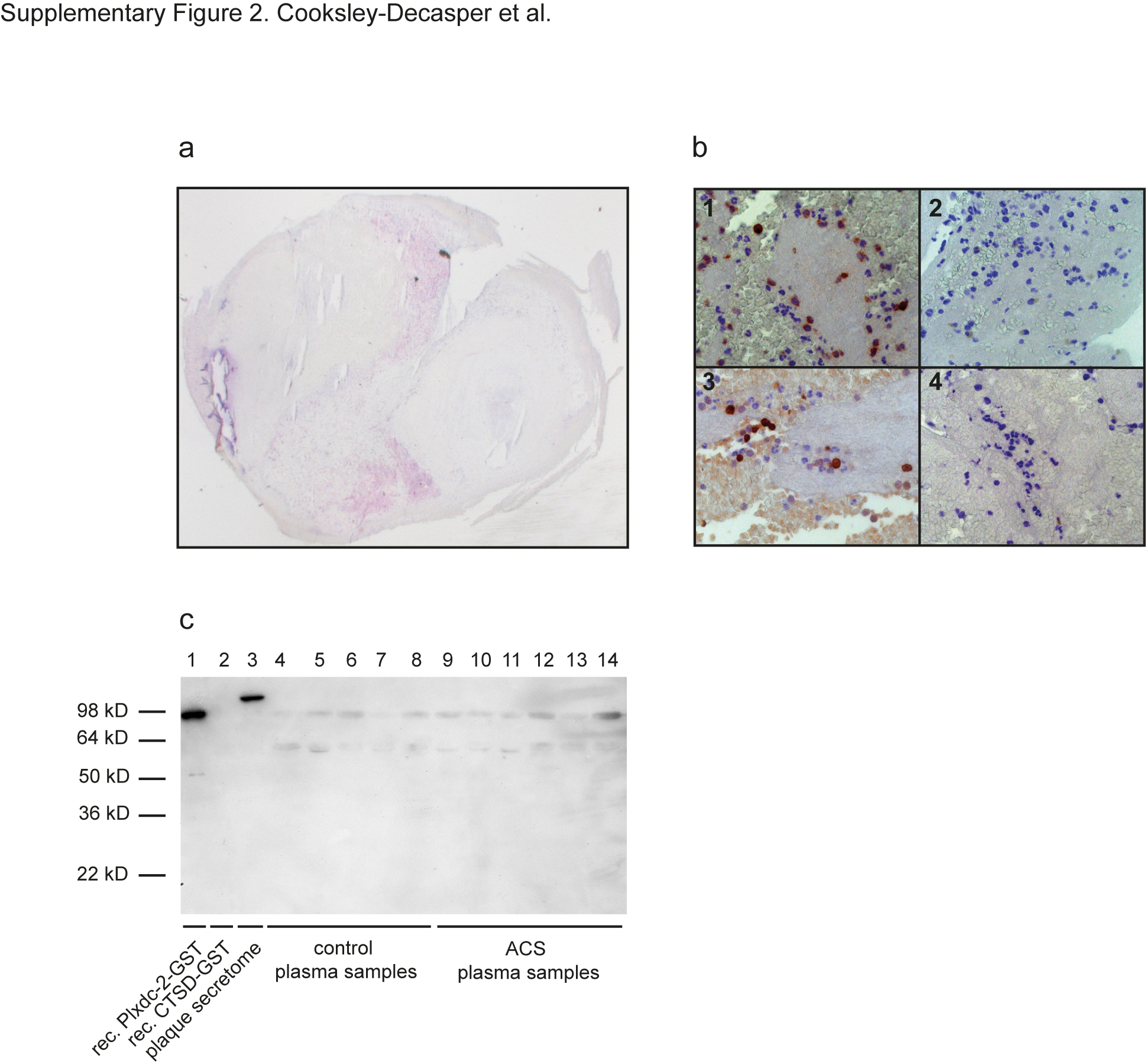


**Supplemental Figure S2. Detection of Plxdc2 in endartectomised tissue and coronary thrombi by immunohistochemistry, and in plasma samples by immunoblotting**. a) Immunohistochemical detection of Plxdc2 in endarterectomised tissue. Plxdc2 is weakly detected in the atherosclerotic part of the tissue. b) Thrombi from two patients (patient 1: upper pictures, and patient 2: lower pictures) were stained with the commercial anti-Plxdc2 mAb 4G10 (1 and 3). Negative control stainings, representing the reactivity of the secondary antibody only, do not show any immunoreactivity (2 and 4). Cells in which Plxdc2 was detected are most likely monocytes and macrophages. There was no extracellular staining observed. c) Detection of Plxdc2 by immunoblotting. Recombinant GST-tagged Plxdc2 (81 kD, lane 1) and a plaque secretome (lane 3) were used as positive controls, recombinant cathepsin D (lane 2) was used as the negative control. Five control and six ACS plasma samples were run on an SDS-PAGE gel, transferred to nitrocellulose membranes and probed with anti-Plxdc2 mAb 4G10. A 56 kD protein, which corresponds to the theoretical molecular weight of Plxdc2, as well as a band of approximately 80 kD, were detected several plasma samples. In plaque secretome, a protein band of around 120 kD was clearly detected.
