# Supplementary material for: Antibody Phage Display Assisted Identification of Junction Plakoglobin as a Potential Biomarker for Atherosclerosis
Source: PLoS One. 2012 Oct 24;7(10):e47985. doi: 10.1371/journal.pone.0047985 (PMC3480477; doi:10.1371/journal.pone.0047985)
Supplement: Figure S3 — Alignment of the amino acid sequences of JUP and of the JUP variant encoded by cDNA FLJ60424. (DOC) [file pone.0047985.s003.doc]

**
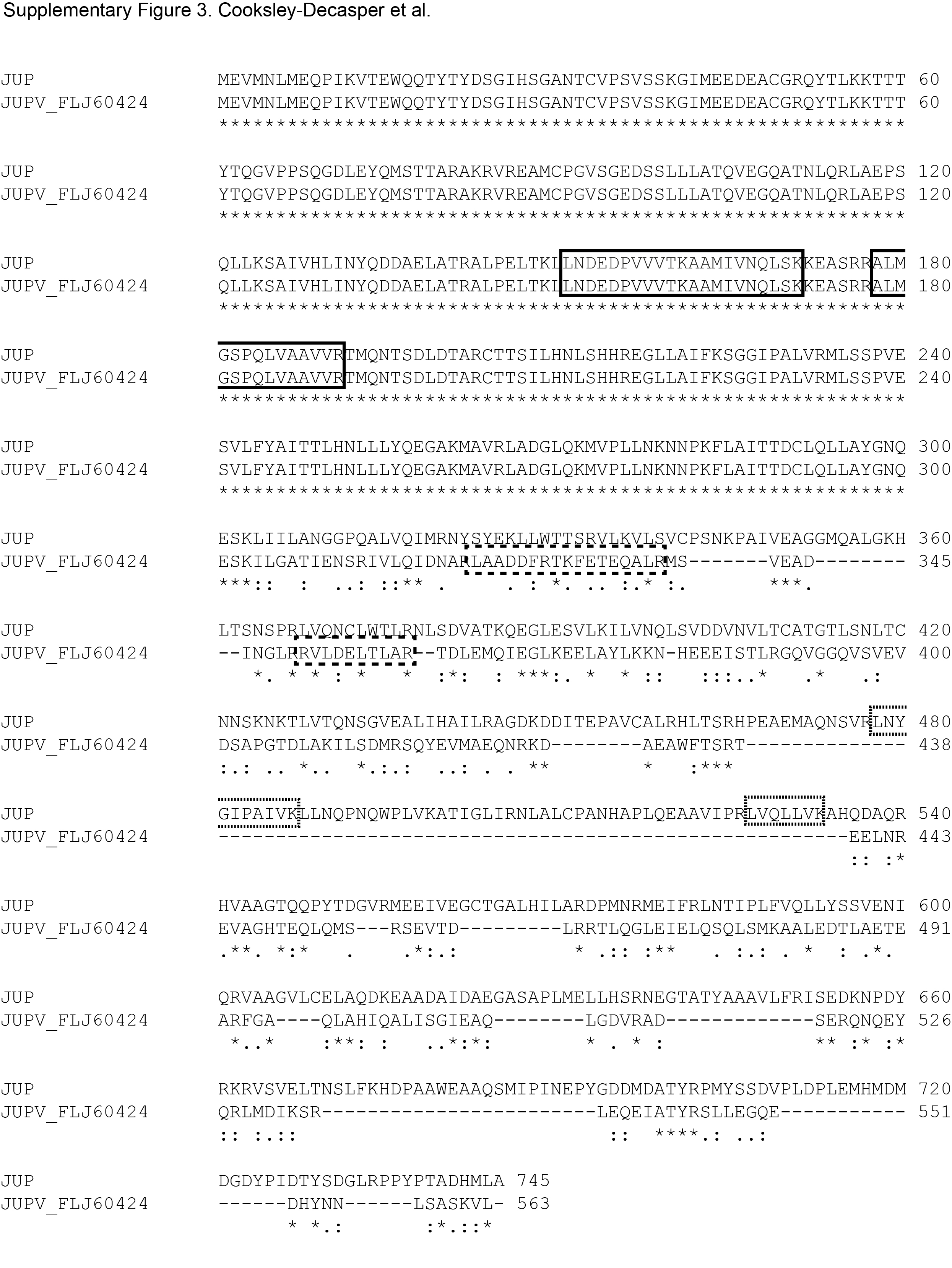
**

**Supplementary Figure S3. Alignment of the amino acid sequences of JUP and of the JUP variant encoded by cDNA FLJ60424.** The N-terminal part (the first 303 amino acids) is identical in both proteins. Peptides that were found in the IP/MS experiment in which scFv 25G5 was used as bait are indicated. The peptides shown in the solid boxes are found in both proteins, the peptides shown in the narrowly dashed boxes are specific for JUP and the peptides marked in the largely dashed boxes are only present in the JUP variant that is encoded by cDNA FLJ60424. The epitopes of the anti-JUP antibodies 2C9 and the scFv 25G5 are unknown.
