# Supplementary material for: Antibody Phage Display Assisted Identification of Junction Plakoglobin as a Potential Biomarker for Atherosclerosis
Source: PLoS One. 2012 Oct 24;7(10):e47985. doi: 10.1371/journal.pone.0047985 (PMC3480477; doi:10.1371/journal.pone.0047985)
Supplement: Methods S1 — Provides detailed methodological information. (DOC) [file pone.0047985.s004.doc]

**Supplementary Methods Cooksley-Decasper et al.**

**Preparation of secretome**

Specimens of arteries from 12 different patients were obtained by thrombendarterectomy of carotid or iliac arteries at the Bruderholz Spital Basel. The tissue was characterised into plaque and best preserved part. The plaque tissue was graded on a scale from 1 to 3, 1 standing for initial stage lesion equivalent to Stary Type I – III, 2 for medium stage lesion equivalent to Stary Type IV and V and 3 for advanced stage complicated lesion equivalent to Stary Type VI - VIII . The sections were cut into similar size pieces of about 3x6 mm, washed with PBS and individually incubated for 24 hours at 37 °C in protein-free RPMI medium (RPMI-1640, Sigma Aldrich) containing 100 µg/ml ampicillin (Merck). After the incubation time, the media containing the secreted and washed out proteins was collected, centrifuged and stored at -20°C until further use. In most cases, further tissue was used to prepare fresh frozen embedded sections and fresh frozen hematoxylin stainings. Secretome samples were dialysed with a cut-off of 12 kD against 50 mM NaHCO_3_, containing 0.5 mM dithiothreitol (DTT), or against PBS at 4°C with three buffer changes, and the protein concentrations were determined with DC low protein concentration microassay (Bio-Rad Laboratories). Six secretomes were used for phage selections, the additional secretomes were used for immunoprecipitations (IPs) and testing of targets.

**Selection of antibodies**

The secretome proteins were biotinylated using EZ-Link Sulfo-NHS-LC-Biotin (Pierce, Thermo Scientific). NHS-LC-biotin stock solution (5 mg/ml) was prepared fresh in 50 mM NaHCO_3_ and 0.5 mM DTT. 50 µl biotin stock solution was added to 1 ml cell extract and incubated for 30 minutes at room temperature (RT). Then, Tris-HCl pH 8.0 was added to a final concentration of 50 mM to stop to the reaction. The biotinylated secretome was then dialysed against 50 mM NaHCO_3_ and 0.5 mM DTT with two buffer changes, followed by additional dialysis against PBS and 0.5 mM DTT with three buffer changes. Glycerol was added to an end concentration of 20% and the biotinylated secretome was stored in small aliquots at -80°C. The uniformity of biotinylation of proteins of all molecular sizes was confirmed on Western blot with detection using avidin coupled to horse radish peroxidase (HRP) and standard development using electrochemiluminescence (ECL) plus western blotting detection kit (Amersham).

A large synthetic human phage-displayed single chain variable fragment (scFv) library, ETH-2-Gold , containing 3 billion individual antibody clones, was amplified as described. Before the selections, the library was depleted of clones binding to some of the most common plasma proteins, including apo-transferrin (Sigma Aldrich), holo-transferrin (Merck), immunoglobulin G (purified from serum using Protein G Dynabeads (Invitrogen)), fibrinogen and complement component 3 (both AbD Serotec). In this library, the scFv antibodies are C-terminally fused with a myc-tag for detection.

Six selections were done with two subtractive selection rounds each. Each selection was performed with secretome from a different patient. Subtraction was achieved by first removing phages binding to control secretome (i.e. secretome produced with the best preserved part of the tissue). The remaining, unbound phages were subsequently panned against plaque secretome from the same patient. First, the phages were incubated with 1 µg of biotinylated control secretome for one hour. Then, 150 µl of magnetic streptavidin beads (Dynabeads, Invitrogen), which were blocked in PBS containing 5% non-fat dried milk powder (MPBS) for one hour, were added and incubate for another 30 minutes. Beads were magnetically separated from the solution and discarded, whereas the solution containing the remaining phages was transferred to a clean tube. This subtraction step was repeated once. Subsequently, a competition step was included, in which phages were incubated with 100 µg of un-biotinylated control secretome for one hour. Then, 1 µg of biotinylated plaque secretome was added and rotated for an additional hour. 150 µl of pre-blocked magnetic streptavidin beads were added and incubated for 15 minutes. Beads were magnetically harvested from the solution and washed several times with PBS containing 0.05% Tween-20 (PBST) and three times with PBS. Bound phages were eluted after the first round of selection with 300 µl 50 mM DTT rotating for 10 minutes. The eluted phages were amplified using trypsin-sensitive helper phage in *Escherichia coli* (*E.coli*) TG1, and purified from the medium by polyethylene glycol precipitation as described previously . In subsequent rounds of selection the phages were eluted by incubating the washed beads with 300 µl of trypsin (Sequencing grade, Promega) (10 g/l in PBS) for 30 minutes at RT. Beads were harvested and discarded, whereas the solution containing the eluted phages was transferred to a clean tube containing 300 µl of fetal calf serum (PAA Laboratories GmbH) to inhibit trypsin activity. Polyclonal phage pools of each round were screened in an enzyme-linked immunosorbent assay (ELISA) for reactivity with control and plaque secretome.

**Screening of single clones**

Single colonies were picked into two round-bottom 96-well plates (Nunc) using sterile toothpicks. They were picked into one 96-well master plate containing 2xTY, 100 µg/ml ampicillin and 2% glucose, and into two 96-well induction plates containing 100 µl 2xTY, 100µg/ml ampicillin and 0.05% glucose. All plates were incubated in a wet chamber for several hours at 37°C and 200 rpm until medium became turbid. To the master plate glycerol was added to a final concentration of 15%, the plate was covered, wrapped in parafilm and stored at -20°C. To the phage induction plate, helper phage was added and the plate was incubated for 40 minutes at 37°C without shaking. Then, 2xTY, containing ampicillin and kanamycin (to give a final kanamycin concentration of 70 µg/ml) was added and incubated overnight at 30°C and 200 rpm to produce phages. To the scFv induction plate, isopropyl β-D-1-thiogalactopyranoside (IPTG) was added to a final concentration of 1 mM, when the medium had become turbid. The plate was then incubated overnight at 30°C and 200 rpm. The next day, the plates were centrifuged at 3’000 rpm for 10 minutes at RT and the supernatant containing the phages or scFv was used for further analysis. Monoclonal phages were analyzed for their reactivity with control and plaque secretomes by ELISA. The scFv expression levels were analyzed by dot blotting. Finally, the cDNA encoding the scFvs of interest were sequenced with primer PelBback (AGCCGCTGGATTGTTATTAC) at Microsynth Services.

**Production of soluble scFv**

ScFv fragments were C-terminally tagged with a 6xHis-tag and VSV-G-tag by cloning the cDNA in the pUC119 vector via compatible NcoI and NotI digestion. ScFv production was induced with addition of IPTG to the culture, which was then grown for 3 hours at 250 rpm. Then, the *E.coli* periplasmic fraction was isolated by sucrose extraction and, if necessary, dialysed against PBS with three buffer changes at 4°C.

Expression levels of scFvs were analyzed by spotting 50 µl of a dilution series on a nitrocellulose membrane, which was subsequently blocked for one hour with MPBS. The scFvs were detected using protein-A antibody conjugated with HRP (Bio-Rad), diluted 1’000-fold in MPBST, followed by development using ECL.

**Immunoprecipitation**

All antibodies for the IPs were produced freshly from bacterial culture. Proteins were precipitated from mixtures of two plaque and control secretomes, using Dynabeads for His-tagged proteins (Invitrogen). 1 ml dialysed, scFv-containing periplasmic fraction was added to the beads and incubated for 15 minutes rotating at RT. The beads were magnetically harvested and washed. Subsequently, secretomes containing 0.14 mg of protein were added to the beads and incubated overnight at 4°C. The next day, the beads were precipitated from the unbound secretome fraction by using a magnet. The supernatant was kept for mass spectrometry (MS) analysis. The beads were washed and the bound proteins were eluted by addition of 200 µl 0.1 M glycine pH 2 and incubation of 15 minutes at RT with regular vortexing. The eluted proteins were separated from the beads magnetically and transferred to a clean tube containing 40 µl of 2 M Tris. The elution step was repeated and the eluted fractions combined. Both the precipitated and the supernatant fraction were stored at -20°C until further analysis.

**Protein identification by mass spectrometry**

All MS experiments were done at the Functional Genomics Center Zurich (FGCZ), a service centre of the University of Zurich. Multiple sample work-up methods were compared, namely 1) in solution digestion of proteins versus separation on SDS-PAGE gels, and 2) reduction, alkylation and digestion by hand versus use of a liquid handling robot. Direct in solution digestion of the precipitated samples resulted in only about 50% of the protein identifications that were obtained with the SDS-PAGE approach. It was furthermore found that combining separation of the proteins on SDS-PAGE and reduction, alkylation and digestion of the proteins executed by a liquid handling robot led to the most protein identifications, compared to work-up by hand.

The samples were concentrated in a speedvac system (Savant) and dissolved in Laemmli buffer . Afterwards the samples were run on Novex 12% Tris-Glycine 1 mm gels (Invitrogen) and stained with RotiBlue (Roth). Each lane was cut into 12 segments and put into individual wells of 96-well plates. All further sample work-up was executed on a liquid handling robot (TECAN). All gel pieces were subjected to reduction of disulfide bridges with DTT, alkylation of cysteines using iodoacetamide and in-gel trypsin digestion for three hours. The samples were then concentrated in a speedvac system and purified using ZipTip-µ C18 (Milipore). Afterwards, the samples were pooled, concentrated and stored dry at -20°C until MS analysis. For MS measurement, the samples were dissolved in 3% acetonitrile (ACN), 0.1% formic acid (FA).

Samples were analyzed on an LTQ-Orbitrap XL mass spectrometer (Thermo Fischer Scientific, Bremen, Germany) coupled to an Eksigent-Nano-HPLC system (Eksigent Technologies, Dublin (CA), USA). Solvent composition at the two channels was 0.2% FA, 1% ACN for channel A and 0.2% FA, 80% ACN for channel B. Peptides were loaded on a self-made tip column (75 µm × 80 mm) packed with reverse phase C18 material (AQ, 3 μm 200 Å, Bischoff GmbH, Leonberg, Germany) and eluted with a flow rate of 200 nl/min by a gradient from 0 to 35% of B in 47 minutes, 95% B in 60 minutes, followed by washing and reconditioning of the column.

Full-scan MS spectra (300-2’000 *m*/*z*) were acquired with a resolution of 60’000 at 400 *m*/*z* after accumulation to a target value of 500’000. Collision induced dissociation MS/MS spectra were recorded in a data-dependent manner in the ion trap from the six most intense signals above a threshold of 500, using a normalized collision energy of 35% and an activation time of 30 ms. Charge state screening was enabled and singly charged states were rejected. Precursor masses already selected for MS/MS were excluded for further selection for 120 s and the exclusion window was set to 20 ppm. The size of the exclusion list was set to a maximum of 500 entries.

The raw files from the mass spectrometer were converted into Mascot generic files with Mascot Distiller and searched against a human-contaminant database (human database including usual protein contaminants) using Mascot Server 2.2. Fixed carbamidomethylation of cysteine and variable oxidation of methionine were set as modifications. The parameters for precursor tolerance and fragment ion tolerance were set to ± 5 ppm and ± 0.8 Da, respectively. Mascot Deamon 2.3.0 (Matrix Science) was used to search the in-house human decoy database . Mascot search parameters were: Tol: 5ppm, Itol: 0.8Da, PFA: 1, Mods: C, Carbamidomethyl, Cle: Trypsin, Search: MIS, Charge: +2 and +3, IT-Mods: M,Oxidation. Data interpretation was done using Scaffold 3 (Proteome Software) with the following parameters: Minimum Protein ID Probability: 99%, Minimum Nr of peptides: 2, Minimum peptide ID probability: 95%.

**Plasma samples and thrombi**

Plasma samples were collected from male patients with ACS, patients with stable CAD and from healthy, angiographically confirmed CAD-free controls . Criteria for study group assignment were a) no angiographically identifiable stenosis (<20%) or diffuse wall sclerosis for the control group, b) at least 1 stenosis ≥ 75% in either left, circumflex or right coronary artery for the CAD group and c) either a ST-elevation (STEMI, typical chest pain and 2 ST segment elevations ≥ 0.1mV) or a non-ST-elevation (NSTEMI, typical chest pain with ≥ 4-fold elevation of Troponin T) myocardial infarction, both being admitted for percutaneous coronary intervention, for the ACS group. Patients with a history of ACS less than six months prior to the study as well as individuals suffering from an active neoplastic, infectious or autoimmune disease were excluded.

Coronary thrombi were obtained by percutaneous coronary interventions of two patients suffering from a STEMI.

**Detection and quantification of JUP and other candidate proteins by immunoblotting**

Prior to SDS-PAGE and immunoblotting, albumin and immunoglobulins were removed from human plasma samples with depletion columns (Qproteome columns, Invitrogen). Thrombi were fragmented, directly dissolved in SDS-PAGE loading buffer containing SDS and DTT and heated for 5 minutes at 95°C. Differentiated macrophages were harvested and lysed in lysis buffer (50 mM HEPES pH 8.0, 1 mM EDTA and 0.2% Triton X-100). Proteins of pre-treated plasma, thrombi and cultured macrophages were separated on polyacrylamide gels by SDS-PAGE and transferred to nitrocellulose by semi-dry blotting. Membranes were then blocked with MPBST. JUP was detected with scFv antibodies 25G5 via the VSV-G-tag, or with anti-JUP monoclonal antibodies (mAb) 2C9 from Lifespan Biosciences (Seattle, USA). The latter antibody was later replaced by 2G9. Comparison of the two antibodies on Western blots containing plasma samples and secretomes demonstrated an identical detection pattern. Anti-VSV-G mAb was purchased from ModiQuest (Nijmegen, The Netherlands) and horseradish peroxidase (HRP)-labelled rabbit anti-mouse antibodies from Dako (Glostrup, Denmark). As a positive control, GST-tagged recombinant JUP from Abnova (Taipei City, Taiwan) was used. As loading control for cell lysates, GAPDH and β-actin were detected with a monoclonal antibody purchased from Abnova and Sigma-Aldrich (Saint Louis, USA), respectively.

Intensities of anti-JUP-immunoreactive bands on developed films were semi-quantitatively measured with ImageJ (National Institutes of Health ). To compare signals of different Western blots, one reference plasma sample was run on each gel for standardization. The intensities of all JUP bands were correlated to the intensity of the anti-JUP-immunoreactive band of this sample (giving relative intensity).

In similar experiments serpin B3 and plexin domain-containing protein 2 (Plxdc2) were immunodetected by using commercial monoclonal mouse antibodies ab55733 from Abcam (Cambridge, England) and 4G10 from Abnova (Taipei City, Taiwan), respectively. Serpin B3 was also detected using the newly generated VSV-G-tagged scFv antibodies 36A8. As positive controls, GST-tagged recombinant serpin B3 and Plxdc2 from Abnova (Taipei City, Taiwan) were used, respectively.

**Immunohistochemistry**

Paraffin-embedded tissue sections of coronary thrombi aspired from culprit coronary arteries of three ACS patients as well as endarterectomized atherosclerotic plaques were pre-treated with 10 mM citrate buffer, pH 6.0, and heated by microwaves at 85-90°C to increase accessibility to the epitopes. The sections were washed in PBS / Tween 0.05% and blocked by successive incubations with 3% H_2_O_2_ (10 minutes at 22°C) and 5% goat serum in PBS (over night at 4°C) to reduce the background. The sections were incubated with the murine anti-JUP mAb 2C9 (IgG2b) or the anti-Plxdc2 mAb 4G10 (IgG kappa). Bound antibodies were detected with biotin-SP-conjugated AffinityPure goat anti-mouse IgG (Jakson ImmunoResearch) and the Vecastain HRP kit (Vector Laboratories, Burlingame CA, USA). Control stainings were performed using mouse IgG 2b or mouse IgG kappa (DakoCytomation) as primary antibodies. Four to six μm serial frozen sections of plaques were cut and fixed in cold acetone for 15 minutes and air dried. Immunohistochemistry was performed by the automated immunostainer Leica Bond-Max (Leica Microsystems AG, Heerbrugg, Switzerland). Slides were incubated by the primary antibody for 15 minutes and the signal visualized by the polymer refine red detection according to the manufactures protocol (Leica Microsystems AG, Heerbrugg, Switzerland). Control stainings were performed using only the secondary antibody. For primary antibodies, anti-JUP monoclonal antibodies (mAb) 2C9 from Lifespan Biosciences (Seattle, USA) and anti-CD68 antibodies (Clone PG-M1) from Dako (Baar, Switzerland) were used.

**References**

1. Stary HC (2001) The development of calcium deposits in atherosclerotic lesions and their persistence after lipid regression. Am J Cardiol 88: 16E-19E.

2. Silacci M, Brack S, Schirru G, Marlind J, Ettorre A, et al. (2005) Design, construction, and characterization of a large synthetic human antibody phage display library. Proteomics 5: 2340-2350.

3. Raats J, van Bree N, van Woezik J, Pruijn G (2003) Generating recombinant anti-idiotypic antibodies for the detection of haptens in solution. J Immunoassay Immunochem 24: 115-146.

4. de Wildt RM, Finnern R, Ouwehand WH, Griffiths AD, van Venrooij WJ, et al. (1996) Characterization of human variable domain antibody fragments against the U1 RNA-associated A protein, selected from a synthetic and patient-derived combinatorial V gene library. Eur J Immunol 26: 629-639.

5. Laemmli UK (1970) Cleavage of structural proteins during the assembly of the head of bacteriophage T4. Nature 227: 680-685.

6. FGCZ_9606 human decoy database, http://fgcz-intranet.uzh.ch/tiki-index.php?page=db_pages.

7. Franzeck FC, Hof D, Spescha RD, Hasun M, Akhmedov A, et al. (2012) Expression of the aging gene p66Shc is increased in peripheral blood monocytes of patients with acute coronary syndrome but not with stable coronary artery disease. Atherosclerosis 220: 282-286.

8. Abramoff MD, Magelhaes, P.J., Ram, S.J. (2004) Image Processing with ImageJ. *Biophotonics International* 11: 36-42.
